# Hydrocele of the Canal of Nuck

**DOI:** 10.5811/westjem.2015.6.27582

**Published:** 2015-10-20

**Authors:** Jagdipak Heer, Rick McPheeters, Asha E. Atwell

**Affiliations:** University of California Los Angeles Kern Medical Center, Department of Emergency Medicine, Los Angeles, California

A 31-year-old gravida 3 Para 3 female with no past medical history, presented to the emergency department complaining of a painless “boil” to the right groin, which had been enlarging for over two months. Although it was generally painless, she did suffer mild dyspareunia at times. Antibiotics prescribed by her primary doctor failed to resolve this mass so she decided to present to the emergency department.

The patient had never experienced any lesion or swelling in that region throughout her life. She denied any prior history of sexually transmitted infections. Her physical examination was unremarkable except for a large (10x5cm) fluctuant mass within the right labia majora (Figure). Her inguinal region had no adenopathy. The mass was non-tender to palpation and was without warmth. The overlying skin was clear without erythema or lesions. It readily transilluminated and upon auscultation, no sounds were noted. Bedside ultrasound confirmed the cystic structure. Because this was inconsistent with a labial or Bartholin’s cyst abscess, consultation with gynecology was obtained. Their leading differential diagnosis was hydrocele of the canal of Nuck. Computed tomography was confirmatory.

Female hydroceles of the canal of Nuck are analogous to scrotal hydroceles in male patients.^1^ They are most common in adult patients and extremely rare in pediatrics, even though the process vaginalis usually obliterates by the first year of life.^2^ Clinical suspicion is encouraged by absence of signs of infections. Ultrasound, computed tomography and magnetic resonance imaging can all help in the diagnosis; however, confirmation is with surgical exploration and pathological examination.^1^ Although extremely rare, fluctuant mass lesions in the female inguinal region should be closely scrutinized for the possibility of a patent canal of Nuck with associated pathology such as a hernia sac, hydrocele or non-communicating cyst.

## Figures and Tables

**Figure:**
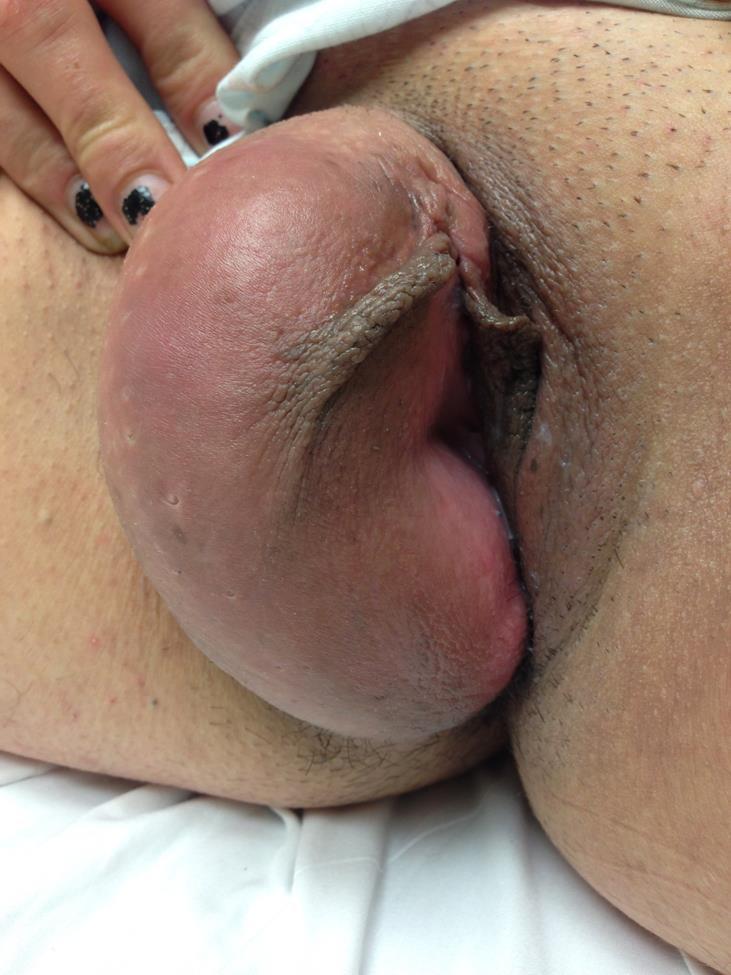
Swelling of the right labia major.
