# This article corrects: “Hydrocele of the Canal of Nuck”

**DOI:** 10.5811/westjem.2015.12.29452

**Published:** 2015-12-17

**Authors:** Jagdipak Heer, Rick McPheeters, Asha E. Atwell, Phillip Aguiniga, Jason Blake

**Affiliations:** * University of California Los Angeles Kern Medical Center, Department of Emergency Medicine, Los Angeles, California; † Kern Medical Center, Department of Emergency Medicine, Bakersfield, California

In the Original Research article entitled “Hydrocele of the Canal of Nuck,” published in the October 2015 issue of the *Western Journal of Emergency Medicine* (2015;16(5):786–787. DOI: 10.5811/westjem.2015.6.27582), there were the following errors in the published article:

1. On page 786, the following authors should have been included: Phillip Aguiniga, Jason Blake. They are research assistants at the Department of Emergency Medicine, Kern Medical Center, Bakersfield, California.

We apologize for this error.

